# Grandine A, a New Proaporphine Alkaloid from the Bark of *Phoebe grandis*

**DOI:** 10.3390/molecules14031227

**Published:** 2009-03-23

**Authors:** Mat Ropi Mukhtar, Ahmad Nazif Aziz, Noel F. Thomas, A. Hamid A. Hadi, Marc Litaudon, Khalijah Awang

**Affiliations:** 1 Centre for Natural Products and Drug Discovery, Block D, Department of Chemistry, Faculty of Science, University of Malaya, 50603 Kuala Lumpur, Malaysia; E-mails: matropi@um.edu.my (M-R.M.), noelfthomas@um.edu.my (N-F.T.), ahamid@um.edu.my (A-A.H.); 2 Institut de Chimie des Substances Naturelles, Centre Nationale de la Recherche Scientifique, UPR2301, 91198, Gif-sur-Yvette, Cedex, France; E-Mail: marc.litaudon@icsn.cnrs-gif.fr (M.L.); 3 Department of Chemical Sciences, Faculty of Science and Technology, Universiti Malaysia Terengganu, 21030 Kuala Terengganu, Terengganu, Malaysia; E-mail: nazif@umt.edu.my (A-N.A.)

**Keywords:** Oxoproaporphine, NMR, *Phoebe grandis*, Aporphine, Dihydrolinearisine.

## Abstract

The stem bark of *Phoebe grandis* afforded one new oxoproaporphine; (–)-grandine A (**1**), along with six known isoquinoline alkaloids: (–)-8,9-dihydrolinearisine (**2**), boldine, norboldine, lauformine, scortechiniine A and scortechiniine B. In addition to that of the new compound, complete ^1^H- and ^13^C-NMR data of the tetrahydroproaporphine (–)-8,9-dihydrolinearisine (**2**) is also reported. The alkaloids’ structures were elucidated primarily by means of high field 1D- and 2D-NMR and HRMS spectral data.

## Introduction

**Figure 1:**
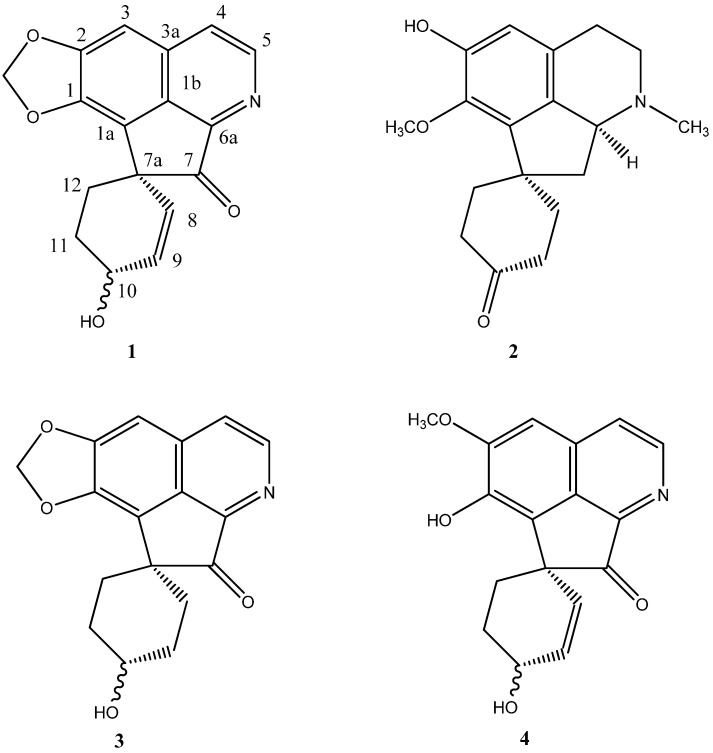
Structures of (+) –grandine A **1**, (-)-8,9-dihydrolinearisine **2**, (+)-scortechiniine B **3** and prooxocryptochine **4. **

The *Phoebe* species (Lauraceae) are producers of isoquinoline alkaloids such as aporphines and oxoaporphines and they are especially known to give proaporphine-tryptamines [1,2,3,4]. At present, only seventeen such dimers have been reported and five were isolated from *Phoebe* [4]. Ten *Phoebe* species have been investigated for their chemical constituents [5,6,7,8,9,10,11] and interestingly only two Malaysian species (*P. grandis* and *P. scortechinii*) produce the proaporphine-tryptamine dimers. In this communication, we report the isolation and characterization of one new oxoproaporphine; (+)-grandine A (**1, **Figure 1) and the complete ^1^H- and ^13^C-NMR spectroscopic data of (–)-8,9-dihydrolinearisine (**2**, Figure 1). In addition to these two compounds five other known alkaloids: (–)-boldine [1], (–)-norboldine [1], (+)-lauformine [6], (+)-scortechiniine A [11] and (+)-scortechiniine B **3 **(Figure 1) [11] were also isolated from the bark of *Phoebe grandis*. 

## Results and Discussion

The alkaloid grandine A (**1**), ${\left[\alpha \right]}_{D}^{23}$ + 55^o^ (c= 1.0, CHCl_3_), was isolated as a brownish amorphous solid. The HRESI^+ ^mass spectrum showed an [M+Na]^+^ peak at m/z 318.0768, thus suggesting a molecular formula of C_17_H_13_NO_4 _(calc. 318.0742 for C_17_H_13_NO_4_Na). Two major fragmentation peaks were observed at m/z 277 and m/z 249, which may be attributed to the loss of a water molecule, [M-H_2_O]^+^, and a carbonyl, [M-CO]^+^, respectively. The IR spectrum revealed absorption bands at 3,376 and 1,729 cm^-1^ due to the OH and the C=O stretching vibrations respectively. The ^1^H-NMR spectrum (Table 1) showed a pair of doublets (*J =* 5.4 Hz) typically found in oxoaporphines [9]. These signals are attributable to H-4 (δ 7.60) and H-5 (δ 8.68), respectively. A singlet ascribable to H-3 appeared at δ 7.19. Two broad singlets representing the methylenedioxy protons were apparent at δ 6.16 and 6.12, respectively. The respective protons in scortechiniine B **3**, which lacks the C-8,9 double bond, gave a singlet at δ 6.13 corresponding to both protons (Table 1). The deshielded aliphatic proton, H-10, resonated as a broad singlet at δ 4.45. The olefinic protons, H-8 and H-9, resonated at δ 5.43 and 6.14 as a doublet and a doublet of doublets, similar to the resonances of the same protons in prooxocryptochine (**4**) (Figure 1, Table 1). However, H-8 appeared as a doublet in the former as compared to doublet of doublets in the latter. 

**Table 1:** 1H-NMR (400 MHz) and ^ 13^C-NMR (100 MHz) spectral data of compounds **1, 3 **and **4** in CDCl_3_ (*δ* in ppm, *J* in Hz) .

Alkaloids		**1**				**3**^a^	**4**^b^
Position	^13^C	δ ^1^H (*J* Hz)	HMBC	NOESY	^13^C	δ ^1^H (*J* Hz)	δ ^1^H (*J* Hz)
1	154.59				154.43		
1a	118.11				120.15		
1b	136.78				136.39		
2	142.62				142.06		
3	99.76	7.19 *s*	1, 2, 3a	OCH_2_O, H-4	99.66	7.04 *s*	7.15 *s*
3a	133.11				133.18		
4	121.66	7.60 *d* (5.4)	3a	H-5	121.58	7.56 *d* (5.4)	7.67 *d* (5.6)
5	146.92	8.68 *d* (5.4)	3a, 4, 6a	H-4	146.96	8.66 *d* (5.4)	8.77 *d* (5.6)
6a	150.86				151.23		
7	204.37				206.02		
7a	52.73				50.57		
8	126.28	5.43 *d* (10.0)	7a,10	H-9	29.38	1.85 *m* (1H) 2.08 *m* (1H)	5.49 *dd* (10, 3.2)
9	134.75	6.14 *dd* (10.0, 2.9)	7a, 10	H-8, H-10	29.92	2.08 *m*	6.25 *dd* (10, 3.2)
10	65.05	4.45 *br s*		H-9, H-11	68.44	3.96 *m*	4.44 *m*
11	28.14	2.38 *m* 2.12 *m*	10, 12	H-12	29.92	2.08 *m*	2.29 *m*
12	28.60	2.12 *m* (2H)	7, 7a, 11	H-11	29.38	1.85 *m*	2.06 *m* 2.26 *m*
OCH_2_O	102.37	6.16 *br s* 6.12 *br s*	1, 2		102.11	6.13 *s*	
2-OMe							4.02 *s*

^a ^^1^H- and ^13^C-NMR Data are reproduced from Mukhtar *et al*. [11]; ^b 1^H-NMR data is reproduced from Wu *et al*. [17].

The ^13^C-NMR spectrum of grandine A (**1**) showed the presence of 17 carbons, which is in agreement with the molecular formula. The resonance of the quaternary spiro carbon, C-7a, at δ 52.7 implied the proaporphinic nature of compound **1**. The C-7 carbonyl peak was observed at δ 204.4. The carbon bearing the hydroxyl group, C-10, resonated at δ 65.1. Thorough analysis of the COSY, HMQC, HMBC and NOESY spectrums allowed the complete assignments of all protons and carbons of grandine A **1** (Table 1). 

(–)-8,9-Dihydrolinearisine (**2**), ${\left[\alpha \right]}_{D}^{23}$ –50.0^o^ (*c* =0.1, MeOH) was isolated as a white amorphous solid. The HRESI^+^ mass spectrum showed the pseudomolecular ion peak, [M+H]^+^, at m/z 302.1707 (calc. 302.1756) thus indicating a molecular formula of C_18_H_23_NO_3_. The EIMS revealed two major fragments at m/z 300 [M-H]^+^ and at m/z 258, [M-CH_2_NCH_3_]^+^, indicative of an *N*-methylproaporphine or an *N*-methylaporphine skeleton [12,13]. The IR spectrum showed a strong carbonyl absorption at 1,712 cm^-1^. In addition, a broad absorption band at 3,345 cm^-1^ was attributed to the presence of a hydroxyl group.

The ^1^H-NMR spectrum (Table 2) displayed one methoxyl singlet at δ 3.76. In addition, one proton singlet was observed at δ 6.41, which may be ascribed to H-3. This observation also indicated that C-2 is substituted. The *N*-methyl group resonated at δ 2.38 and the aliphatic protons gave a multiplet between δ 1.82 to δ 3.27. H-6a resonated at δ 3.23 (dd, *J, J’* = 11.0, 6.3 Hz) while H-7α appeared as a doublet of doublets at δ 2.72. 

**Table 2:** 1H-NMR (400 MHz) and ^ 13^C-NMR (100 MHz) spectral data of compounds **2 **in CDCl_3_ plus two drops of CD_3_OD (*δ* in ppm, *J* in Hz).

Position	δ ^13^C	δ ^1^H (Hz)	DEPT	HMBC
1	142.83			
1a	137.99			
1b	127.11			
2	149.84			
3	114.32	6.41 *s*	CH-3	1, 1b, 2, 4
3a	131.83			
4	26.72	α 2.61 *m*	CH_2_-4	1b, 5
		β 2.55 *m*		
5	54.76	α 2.38 *m*	CH_2_-5	3a, 6a, N-CH_3_
		β 3.07 *m*		
6a	65.12	3.23 *dd*	CH-6	1b, 7, 7a
		(11.0, 6.3)		
7	42.21	α 2. 72 *dd* (11.0, 6.3)	CH_2_-7	6a, 1b, 7a
		β 1.65 *dt* (11.0)		6a, 7a, 8, 12
7a	47.29			
8	33.70	ax, 2.22 *m*	CH_2_-8	7
		eq, 1.98 *m*		
9	38.58	2.48 *m*	CH_2_-9	8, 10
10	212.20			
11	39.13	ax, 2.48 *m*	CH_2_-11	10, 12
		eq, 2.80 *m*		
12	36.23	ax, 2.82 *m*	CH_2_-12	7a, 7
		eq, 1.82 *m*		
1-OCH_3_	61.00	3.76 *s*		1
*N*-CH_3_	43.06	2.38 *s*		5, 6a

The ^13^C-NMR spectrum of **2** showed the presence of eighteen carbon atoms; two methyls, seven methylenes, two methines, one carbonyl carbon and six quarternary carbons. The quaternary C-7a resonated at δ 47.3, while the C-1 methoxyl group peak appeared at δ 61.0. The NOE differential experiment showed signal enhancements of H-7α and H-8eq upon irradiation of H-6a, therefore indicating that H-6a is *syn* to H-7α and H-8eq [11]. Irradiation of H-3 resulted in the enhancement of H-4 (δ 2.55), thus suggesting that the methoxyl group is attached to C-1. (–)-8,9-Dihydrolinearisine (**2**) adopts an *S* configuration at C-6a, based on its negative optical rotation value ${\left[\alpha \right]}_{D}^{23}$ –50.0^o^ [12]. Alkaloid **2** is actually the enantiomer of (+)-*N*-methyltetrahydrocrotonosine, which occurred in *Croton* species [14]. 

## Conclusions

In summary, we have observed that *Phoebe grandis* produces alkaloids similar to those of *Phoebe scortechinii* [2,3]. Both plants yielded proaporphines, aporphines and proaporphine-tryptamines. Earlier work on the barks of *Phoebe grandis*, collected from the northern part of Peninsular Malaysia, had indicated only aporphine alkaloids [1] while another study on the same plant species collected from Pahang, on the east coast of Peninsular Malaysia, has shown the presence of aporphines and proaporphines. This could be due to either seasonal variations or a difference in the soil types (the former was collected from a lowland area while the latter was collected from a highland area). The occurrence of the new proaporphine in *Phoebe grandis* is of special interest, in view of the fact that this type of alkaloid is a precursor of aporphines [15, 16] and proaporhine–tryptamines, found in *Phoebe* species. To the knowledge of the authors, only two oxoproaporphines have been previously reported; scortechiniine B (**3**) [11] and prooxocryptochine (**4**) [17]. Grandine A (**1**) and scortechiniine B (**3**) occurred in the *Phoebe* species, while prooxocryptochine (**4**) was isolated from the wood of *Cryptocarya chinensis* [17]. Incidently, both *Phoebe* and *Cryptocarya* belong to the family Lauraceae.

## Experimental

### General

The optical rotations were recorded on s Jasco (Japan) P1010 instrument equipped with a tungsten lamp. HRMS was obtained on a Thermo Finnigan Automass Multi. The ultraviolet spectra were obtained in MeOH on a Shimadzu UV-160A ultraviolet-visible spectrometer. The infrared spectra were taken on a Perkin Elmer 1600 Double-Beam recording spectrometer, using chloroform as solvent. The ^1^H-NMR and ^13^C-NMR spectra were recorded in deuterated chloroform on a JEOL 400 MHz (unless stated otherwise); chemical shifts are reported in ppm on δ scale, and the coupling constants are given in Hz. Silica gel 60, 70-230 mesh ASTM (Merck 7734) and silica gel 60, 230-400 Mesh ASTM (Merck 9385) were used for column and flash chromatography, respectively. Mayer’s reagent was used for alkaloid screening. 

### Plant material

*Phoebe grandis* (Lauraceae), collected in May 2001 from Kuala Tahan Forest Reserve, Pahang, Malaysia was identified by Mr Teo Leong Eng. A voucher specimen (KL 4994) is deposited at the Herbarium of Department of Chemistry, University of Malaya, Kuala Lumpur, Malaysia and at the Herbarium of the Forest Research Institute, Kepong, Malaysia. 

### Extraction and isolation of the alkaloids

The dried stem bark (1.0 kg) of *Phoebe grandis* was ground and extracted exhaustively with hexane followed by CH_2_Cl_2_ by Soxhlet extractor for 17 hours. Extraction of alkaloids was carried out in the usual manner, which has been described in detail [1, 2] and gave 4.02 g of crude alkaloid. CH_2_Cl_2_-extracted crude alkaloid (1.0 g) was subjected to column chromatography. The isolation and purification of compound **1** and **2** were carried out by chromatography on a small column and preparative TLC (Silica gel 60F_254_) yielding 4.2 mg of grandine A (1) (CH_2_Cl_2_: MeOH, 98:2) and 7.4 mg of (–)-8,9-dihydrolinearisine (**2**) (CH_2_Cl_2_: MeOH, 95:5). 

*Grandine A* (**1**): Isolated as a brown amorphous solid; ${\left[\alpha \right]}_{D}^{23}$ +55^o^ (*c* = 1.0, CHCl_3_); UV: λ_ethanol_: 250, 320 nm; IR ν_max_ (liquid film): 3376, 1927, 2922 cm^-1^; HREIMS *m/z*: [M+Na]^+^, 318.0768 (calc. 318.0742 for C_17_H_13_NO_4_Na); ^1^H- and ^13^C-NMR see Table 1.

*(-)-8,9-Dihydrolinearisine* (**2**): Isolated as a white amorphous solid; ${\left[\alpha \right]}_{D}^{23}$ –50.0 (*c* = 0.1, MeOH); UV λ_ethanol_: 226, 283 and 302 nm; IR ν_max_ (liquid film) : 1672, 3345, 945.4(OCH_2_O) cm^-1^; HRESI^+^ *m/z*: [M+H]^+^ 302.1707 (calc. 302.1756 for C_18_H_24_NO_3_); EIMS: m/z (rel. int. %): 301, 300 (100); ^1^H- and ^13^C-NMR see Table 2.
